# Supplementary material for: Therapeutic Potential of Glutaminase Inhibition Targeting Metabolic Adaptations in Resistant Melanomas to Targeted Therapy
Source: Int J Mol Sci. 2025 Aug 25;26(17):8241. doi: 10.3390/ijms26178241 (PMC12428566; doi:10.3390/ijms26178241)
Supplement: Supplementary file 1 [file ijms-26-08241-s001.zip › ijms-3728275-supplementary.pdf]

Supplementary Materials

Table S1: Primer sequences for RTqPCR conducted to quantify mRNA levels of GAC, KGA, GLS2, GLUD1, SLC1A5, SLC7A5, SLC38A2, PPARGC1A, TFAM, PPRC1, NRF2, CAT, GSS, GSR1, GPX1, and 18S as a housekeeping gene.

Table S2: List of completed or active trials studying the toxicity and efficacy of CB-839 on cancer (https://clinicaltrials.gov/study/), the clinical study number about metastatic melanoma is in bold.

Figure S1: Relative glutamine/glutamate ratio assessed in sensitive and resistant melanoma cells to RTKi/MAPKi. A. Glutamine/glutamate ratio was evaluated after glutamine and glutamate quantification in the intracellular medium of sensitive and resistant cells exposed to RTKi/MAPKi (1 μM) for 24 hours.

Figure S2: Quantification of the immunofluorescence analysis of NRF2 protein shown in Figure 2E. Pixel counts of green fluorescence were measured in the cytoplasmic (Cyto) and nuclear (Nuc) compartments of sensitive (S) and resistant (R) cell lines. Red = MM074, Yellow = MM161, Blue = HBL cells.

Figure S3: Effect of α-KG supplementation (5 mM) on the antiproliferative effect (crystal violet staining) induced by CB-839 (10 μM) in BRAF mutated MM074 cells for 72 hours, showing that α-KG did not restore proliferation inhibited by CB-839 in these sensitive cells to the BRAF inhibitor.

Figure S4: Progression of animal weight during the in vivo experiment. Weight was monitored for 1 week after cell injection and then for 3 weeks during CB-839 oral gavage treatment (upper panel), comparing untreated mice (blue line) with CB-839-treated mice (orange line). Box plots show animal weights on days 23 and 27 for untreated/control (CTR) and CB-839-treated groups (lower panel).

Table S1

Gene	Protein	Forward sequence	Reverse sequence
18S	18S	CATTTAGGTGACACTATAGAAGACGATCAGATACCGTCGTAGTTCC	GGATCCTAATACGACTCACTATAGGCCTTTAAGTTTCAGCTTTGCAACC
GLS1	KGA	GGTCTCCTCCTCTGGATAAGATGG	CCCGTTGTCAGAATCTCCTTGAGG
GLS1	GAC	GGTCTCCTCCTCTGGATAAGATGG	GATGTCCTCATTTGACTCAGGTGAC
GLS2	GLS2	GGCCATGTGGATCGCATCTT	ACAGGTCTGGGTTTGACTTGG
GLUD1	GDH	GGATTCTAACTACCACTTGCTC	GAACGCTCCATTGTGTATGC
SLC1A5	SLC1A5	TCATGTGGTACGCCCCTGT	GCGGGCAAAGAGTAAACCCA
SLC7A5	SLC7A5	CCGTGAACTGCTACAGCGT	CTTCCCGATCTGGACGAAGC
SLC38A2	SLC38A2	ACCGCAGCCGTAGAAGAATG	GCCAGACGGACAATGAGAAGAA
PPARGC1A	PGC1α	TCTGAGTCTGTATGGAGTGACAT	CCAAGTCGTTCACATCTAGTTCA
TFAM	TFAM	TCCCCCTTCAGTTTTGTGTA	ATCAGGAAGTTCCCTCCAAC
PPRC1	PPRC1	CACCCACCTGGCATCACTT	ACCTGTCGCACCACAACTG
NRF2	NRF2	TCAGCGACGGAAAGAGTATGA	CCACTGGTTTCTGACTGGATGT
CAT	Catalase	CTCCGGAACAACAGCCTTCT	ATAGAATGCCCGCACCTGAG
GSS	Glutathione synthetase	ATACCATCCTGCGTCACCTG	TCCTTGCCCGCCTCATAGTT
GSR1	Glutathione reductase	ATGATCAGCACCAACTGCAC	ACCTCCTTGACCTGGGAGAA
GPX1	Glutathione peroxydase	CCGGGACTACACCCAGATGA	TCTTGGCGTTCTCCTGATGC

Table S2

NCT Number	Study Title	Study Status	Conditions	Interventions	Sponsor
NCT02944435	A Comparative, Pharmacokinetic Study of CB-839 Capsule and Tablet Formulations in Healthy Adults	COMPLETED	Healthy Volunteers	DRUG: CB-839 Capsules, DRUG: CB-839 Tablets	Calithera Biosciences, Inc
NCT02861300	CB-839 + Capecitabine in Solid Tumors and Fluoropyrimidine Resistant PIK3CA Mutant Colorectal Cancer	COMPLETED	Colorectal Cancer, Colon Cancer, Rectal Cancer, Solid Tumor	DRUG: CB-839, DRUG: Capecitabine	David Bajor, MD
NCT03965845	A Study of Telaglenastat (CB-839) in Combination With Palbociclib in Patients With Solid Tumors	COMPLETED	Solid Tumors, NSCLC, CRC, KRAS Gene Mutation	DRUG: Telaglenestat (CB-839), DRUG: Palbociclib Oral Capsule or Tablet [Ibrance]	Calithera Biosciences, Inc
NCT02071927	Study of the Glutaminase Inhibitor CB-839 in Leukemia	COMPLETED	Acute Myeloid Leukemia (AML), Acute Lymphocytic Leukemia (ALL)	DRUG: CB-839, DRUG: CB-Aza	Calithera Biosciences, Inc
NCT03163667	CB-839 With Everolimus vs. Placebo With Everolimus in Participants With Renal Cell Carcinoma (RCC)	COMPLETED	Clear Cell Renal Cell Carcinoma	DRUG: Placebo, DRUG: CB-839, DRUG: everolimus	Calithera Biosciences, Inc
NCT03057600	Study of CB-839 in Combination w/ Paclitaxel in Participants of African Ancestry and Non-African Ancestry With Advanced Triple Negative Breast Cancer (TNBC)	COMPLETED	Triple Negative Breast Cancer, TNBC - Triple-Negative Breast Cancer	DRUG: Paclitaxel, DRUG: CB-839	Calithera Biosciences, Inc
NCT03875313	Study of CB-839 (Telaglenastat) in Combination With Talazoparib in Patients With Solid Tumors	TERMINATED	Solid Tumor, Clear Cell Renal Cell Carcinoma, TNBC - Triple-Negative Breast Cancer, Colorectal Cancer, CRC, RCC, ccRCC	DRUG: CB-839, DRUG: Talazoparib	Calithera Biosciences, Inc
NCT02071888	Study of the Glutaminase Inhibitor CB-839 in Hematological Tumors	COMPLETED	Non-Hodgkin's Lymphoma (NHL), Multiple Myeloma, Waldenstrom's Macroglobulinemia (WM), Other B-cell NHL Subtypes, Including WM, T-cell NHL	DRUG: CB-839, DRUG: CB-839 and low dose dexamethasone, DRUG: CB-839, pomalidomide, and low dose dexamethasone	Calithera Biosciences, Inc
NCT03428217	CANTATA: CB-839 With Cabozantinib vs. Cabozantinib With Placebo in Patients With Metastatic Renal Cell Carcinoma	COMPLETED	Advanced Renal Cell Carcinoma, Metastatic Renal Cell Carcinoma	DRUG: CB-839, DRUG: Cabozantinib, DRUG: Placebo	Calithera Biosciences, Inc
NCT03798678	CB-839 HCl in Combination With Carfilzomib and Dexamethasone in Treating Patients With	ACTIVE_NOT_RECRUITING	Recurrent Multiple Myeloma, Refractory Multiple Myeloma	DRUG: Carfilzomib, DRUG: Dexamethasone, DRUG: Telaglenastat Hydrochloride	National Cancer Institute (NCI)

	Recurrent or Refractory Multiple Myeloma				
NCT03047993	Glutaminase Inhibitor CB-839 and Azacitidine in Treating Patients With Advanced Myelodysplastic Syndrome	COMPLETED	Acute Myeloid Leukemia With Multilineage Dysplasia, Blasts 20-30 Percent of Bone Marrow Nucleated Cells, Blasts 20-30 Percent of Peripheral Blood White Cells, Chronic Myelomonocytic Leukemia, High Risk Myelodysplastic Syndrome, IPSS Risk Category Intermediate-2, Myelodysplastic Syndrome	DRUG: Azacitidine, DRUG: Glutaminase Inhibitor CB-839	M.D. Anderson Cancer Center
NCT03944902	CB-839 in Combination With Niraparib in Platinum Resistant BRCA -Wild-type Ovarian Cancer Patients	TERMINATED	Ovarian Cancer, Resistant BRCA Wild-Type Ovarian Cancer	DRUG: Cohort 1: Dose Escalation, DRUG: Cohort 2: Dose Escalation	University of Alabama at Birmingha m
NCT04250545	Testing of the Anti Cancer Drugs CB-839 HCl (Telaglenastat) and MLN0128 (Sapanisertib) in Advanced Stage Non-small Cell Lung Cancer	ACTIVE_NOT_R ECRUITING	Leptomeningeal Neoplasm, Metastatic Lung Non-Small Cell Carcinoma, Metastatic Malignant Neoplasm in the Brain, Recurrent Lung Non-Small Cell Carcinoma, Stage IV Lung Cancer AJCC v8, Stage IVA Lung Cancer AJCC v8, Stage IVB Lung Cancer AJCC v8	DRUG: Sapanisertib, DRUG: Telaglenastat Hydrochloride	National Cancer Institute (NCI)
**NCT03263429**	Novel PET/CT Imaging Biomarkers of CB-839 in Combination With Panitumumab and Irinotecan in Patients With Metastatic and Refractory RAS Wildtype Colorectal Cancer	COMPLETED	Colorectal Cancer, Metastatic Colorectal Cancer, RAS Wild Type Colorectal Cancer, Refractory Colorectal Cancer	DRUG: Glutaminase Inhibitor CB-839, BIOLOGICAL: Panitumumab, DRUG: Irinotecan Hydrochloride (phase I only), OTHER: Laboratory Biomarker Analysis, OTHER: Pharmacological Study, DEVICE: Imaging with 11C-Glutamine PET/CT scans and 18F-FSPG PET/CT scans	Vanderbilt-Ingram Cancer Center

NCT02071862	Study of the Glutaminase Inhibitor CB-839 in Solid Tumors	COMPLETED	Solid Tumors, Triple-Negative Breast Cancer, Non Small Cell Lung Cancer, Renal Cell Carcinoma, Mesothelioma, Fumarate Hydratase (FH)-Deficient Tumors, Succinate Dehydrogenase (SDH)-Deficient Gastrointestinal Stromal Tumors (GIST), Succinate Dehydrogenase (SDH)-Deficient Non-gastrointestinal Stromal Tumors, Tumors Harboring Isocitrate Dehydrogenase-1 (IDH1) and IDH2 Mutations, Tumors Harboring Amplifications in the cMyc Gene	DRUG: CB-839, DRUG: Pac-CB, DRUG: CBE, DRUG: CB-Erl, DRUG: CBD, DRUG: CB-Cabo	Calithera Biosciences, Inc
NCT04824937	Telaglenastat + Talazoparib In Prostate Cancer	UNKNOWN	Prostate Cancer Metastatic	DRUG: Telaglenastat, DRUG: Talazoparib	Massachusetts General Hospital
**NCT02771626**	Study CB-839 in Combination With Nivolumab in Patients With Melanoma, Clear Cell Renal Cell Carcinoma (ccRCC) and Non-Small Cell Lung Cancer (NSCLC)	TERMINATED	Clear Cell Renal Cell Carcinoma (ccRCC), Melanoma, Non-small Cell Lung Cancer (NSCLC)	DRUG: CB-839, DRUG: Nivolumab	Calithera Biosciences, Inc
NCT03528642	Telaglenastat With Radiation Therapy and Temozolomide in Treating Patients With IDH-Mutated Diffuse Astrocytoma or Anaplastic Astrocytoma	ACTIVE_NOT_RECRUITING	Astrocytoma, IDH-Mutant, Grade 2, Astrocytoma, IDH-Mutant, Grade 3	OTHER: Questionnaire Administration, RADIATION: Radiation Therapy, DRUG: Telaglenastat Hydrochloride, DRUG: Temozolomide	National Cancer Institute (NCI)
NCT03831932	Telaglenastat Hydrochloride and Osimertinib in Treating Patients With EGFR-Mutated Stage IV Non-small Cell Lung Cancer	ACTIVE_NOT_RECRUITING	Advanced Lung Non-Small Cell Carcinoma, Metastatic Lung Non-Small Cell Carcinoma, Stage IV Lung Cancer AJCC v8	PROCEDURE: Biospecimen Collection, PROCEDURE: Computed Tomography, PROCEDURE: Magnetic Resonance Elastography, DRUG: Osimertinib, PROCEDURE: Positron Emission Tomography, DRUG: Telaglenastat Hydrochloride, PROCEDURE: X-Ray Imaging	National Cancer Institute (NCI)

NCT03872427	Testing Whether Cancers With Specific Mutations Respond Better to Glutaminase Inhibitor, Telaglenastat Hydrochloride, Anti-Cancer Treatment, BeGIN Study	ACTIVE_NOT_RECRUITING	Advanced Malignant Solid Neoplasm, Metastatic Malignant Solid Neoplasm, NF1 Mutation Positive Malignant Peripheral Nerve Sheath Tumor, Unresectable Malignant Solid Neoplasm	PROCEDURE: Biospecimen Collection, PROCEDURE: Computed Tomography, PROCEDURE: Magnetic Resonance Imaging, OTHER: Pharmacodynamic Study, PROCEDURE: Positron Emission Tomography, DRUG: Telaglenastat Hydrochloride	National Cancer Institute (NCI)
NCT04265534	KEAPSAKE: A Study of Telaglenastat (CB-839) With Standard-of-Care Chemoimmunotherapy in 1L KEAP1/NRF2-Mutated, Nonsquamous NSCLC	TERMINATED	Non-Small Cell Lung Cancer, Non-squamous Non-small-cell Lung Cancer, Non-Squamous Non-Small Cell Neoplasm of Lung, KEAP1 Gene Mutation, NRF2 Gene Mutation, NFE2L2 Gene Mutation	DRUG: Telaglenastat, DRUG: Carboplatin Chemotherapy, DRUG: Pemetrexed Chemotherapy, BIOLOGICAL: Pembrolizumab Immunotherapy, DRUG: Placebo, DIETARY_SUPPLEMENT: Folic acid 400 -1000 Î¼g, DIETARY_SUPPLEMENT: Vitamin B12 1000 Î¼g, DRUG: Dexamethasone 4 mg	Calithera Biosciences, Inc

Figure S1

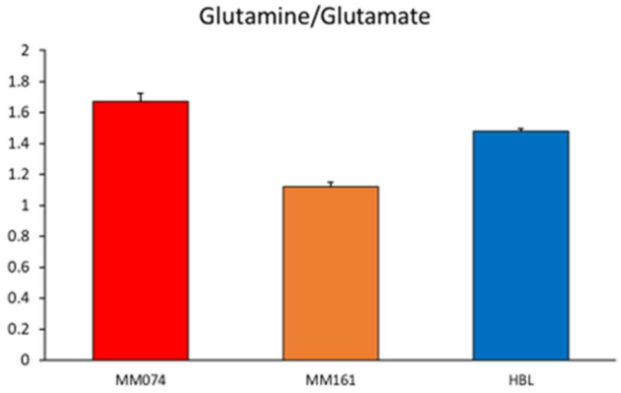
Glutamine/Glutamate
2
1.8
1.6
1.4
1.2
1
0.8
0.6
0.4
0.2
0
MM074
MM161
HBL

Figure S2

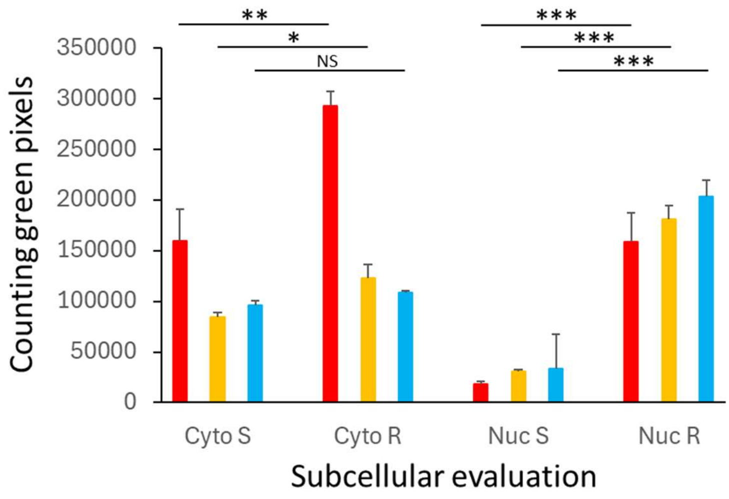

**
*
NS
***
***
***
350000
300000
250000
200000
150000
100000
50000
0
Counting green pixels
Cyto S
Cyto R
Nuc S
Nuc R
Subcellular evaluation

Figure S3

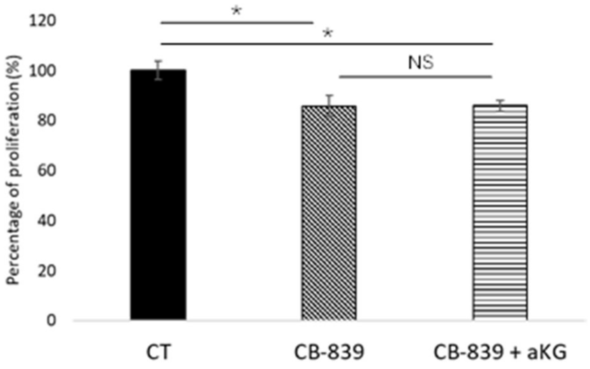
120
100
80
60
40
20
0
Percentage of proliferation (%)
*
*
NS
CT
CB-839
CB-839 + aKG

Figure S4

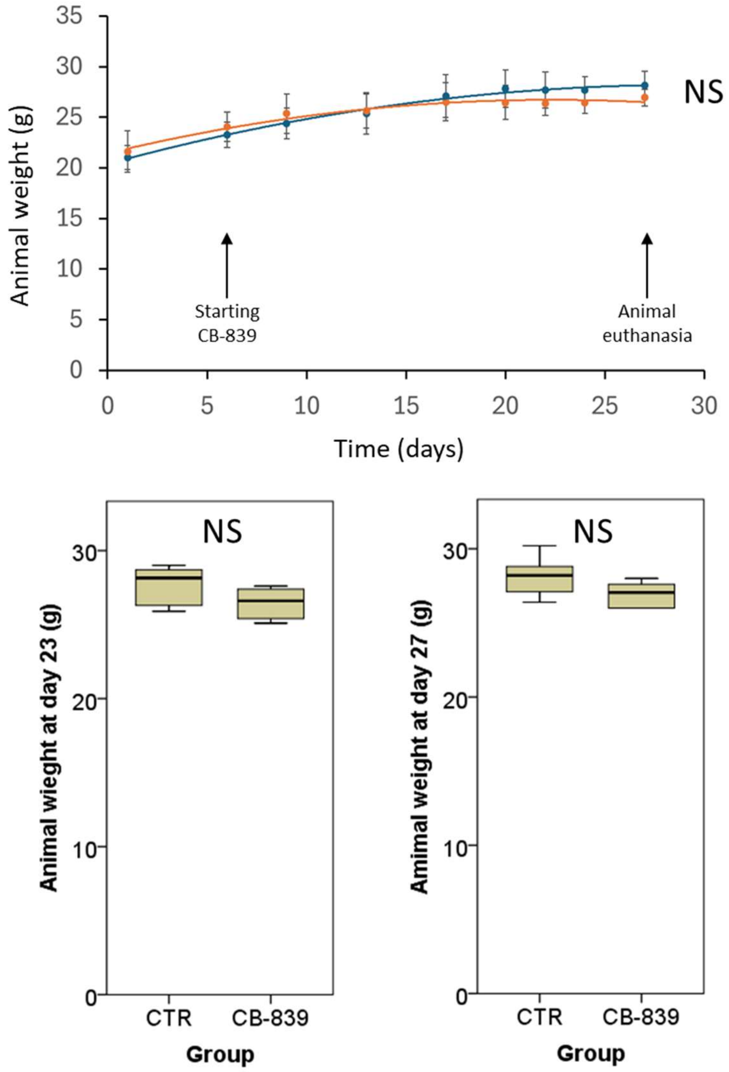

Animal weight (g)
Time (days)
NS
Starting CB-839
Animal euthanasia
Animal wieght at day 23 (g)
Amimal weight at day 27 (g)
CTR
CB-839
Group
